# Copings and suicidal ideation in men from the clinical and control groups

**DOI:** 10.1192/j.eurpsy.2024.1640

**Published:** 2024-08-27

**Authors:** T. I. Medvedeva, O. M. Boyko, O. U. Vorontsova, S. N. Enikolopov

**Affiliations:** ^1^Clinical psychology, Federal Stare Budgetary Scientific Institution “Mental Health Research Center”, Moscow, Russian Federation

## Abstract

**Introduction:**

Suicidal ideation is often an indicator of suicidal risk. However, suicidality is one of the most stigmatized themes thus suicidal ideation can be difficult to diagnose using direct questions. So, it’s impotent to look for psychological traits those may be linked to suicidal ideation. This can be useful for the diagnostic of suicidal risk and prevention of suicidal behavior. The identification of copings that correlate with suicidal ideation and do not depend on mental health allows finding universal ways to reduce suicidal risk.

**Objectives:**

The aim of the study is to find “copings” which have universal impact on suicidal ideation in men.

**Methods:**

The data were obtained using the study of 193 men (clinical group: 67 men with F20, F31, F33 diagnosis aged 17 to 34 (mean age 21,1±4,25); control group: 126 men aged 18 to 63 (mean age 40,04±14,71) who never asked for psychiatric assistance. 3 questions about suicidal ideation (estimate of frequency of last week with Likert’ scale from 0 - “not at all” to 4 -“extremely”), COPE (Carver, 1989). Correlation analysis (Spearman) were used.

**Results:**

The table consists correlation that are statistically significant for both groups.

**Table:** 

Control group					
question	Denial	Behavioral disengagement	Mental disengagement/self-distraction	Substance use	Venting
Feeling hopeless about the future	,218*	,177*	,237**	,208*	,304^**^
Thoughts of ending your life	,189*	,217*	,240**	,189*	---
Thoughts of death or dying	,117	---	,290**	,215*	,301^**^

Clinical group					
Feeling hopeless about the future	,494**	,177*	,478**	,208*	,375^**^
Thoughts of ending your life	,347**	,217*	,363**	,342**	---
Thoughts of death or dying	,353^**^	---	,302*	,323**	,249*

* - p≤0,05; ** p ≤0,01

**Conclusions:**

Our results demonstrate that avoidant copings (Denial, Behavioral disengagement, Mental disengagement /self-distraction) link to suicidal ideation in all men regardless of their mental state. This can be explained by general pathological effect of avoidant copings of people lives: its hinder an identification and settlement of the everyday problems and its contribute to worsening of the situation. This underlines the importance of promotion more active copings as part of prevention antisuicidal work. The “Substance use” coping comforts for a while and anyway helps to formation suicidal ideation through worsening a mental and physical states, increase impulsivity. All this shows that work aimed the substance use prevention constitutes a suicidal ideation prevention work. Increased frequency of use of a Venting coping can leave men in emotional storm state that carries risk of suicide. This points to the importance of wide popularization of affordable ways to the calm, reduce of the power of emotion.

**Disclosure of Interest:**

None Declared

